# Integrating Evidence on Dynapenia and Dynapenic Obesity: An Umbrella Review of Health Outcomes Among Community-Dwelling Older Adults

**DOI:** 10.3390/healthcare14030301

**Published:** 2026-01-26

**Authors:** Shih-Sen Lin, Sung-Yun Chen, Hsiao-Chi Tsai, Shu-Fang Chang

**Affiliations:** 1 Division of Chest Medicine, Department of Internal Medicine, Shin Kong Wu Ho-Su Memorial Hospital, Taipei 111, Taiwan; m008932@ms.skh.org.tw; 2 School of Nursing, National Taipei University of Nursing and Health Sciences, Taipei 112, Taiwan; z1315@tpech.gov.tw; 3 Nursing Department, Taipei City Hospital Renai Branch, Taipei 106, Taiwan; 4 Department of Nursing, Cardinal Tien Hospital, Xindian District, New Taipei City 231, Taiwan

**Keywords:** dynapenia, dynapenic obesity, mortality, fall, multimorbidity, umbrella review

## Abstract

**Background:** Dynapenia refers to the age-related decline in muscle strength that occurs even when muscle mass is preserved. It has become an important issue in older adults because reduced strength is strongly linked to many negative health outcomes. When dynapenia occurs together with obesity—referred to as dynapenic obesity or dynapenic abdominal obesity—the risks, including mortality, falls, and the development of multiple chronic conditions, appear to increase even further. This umbrella review aimed to bring together and summarize existing systematic reviews and meta-analyses that examined how dynapenia and its obesity-related subtypes are associated with mortality, falls, and multimorbidity among community-dwelling older adults. **Methods:** Following PRISMA 2020 and JBI guidelines, six major databases and search engines (PubMed, Embase, Cochrane Library, Scopus, CINAHL, and Airiti Library) were searched from their inception to October 2025. Systematic reviews and meta-analyses involving adults aged 60 years and older and reporting quantitative results on the relationships between dynapenia-related conditions and adverse health outcomes were included. The methodological quality of each review was evaluated using AMSTAR 2, and the certainty of evidence was assessed with the GRADE approach. This umbrella review followed the PRIOR framework and was reported according to PRISMA 2020. The protocol for this review was registered in PROSPERO (ID: CRD 42023415232). **Results:** A total of four systematic reviews and meta-analyses were included, covering more than 73,000 community-dwelling older adults. The pooled data showed that dynapenic obesity significantly increased the risk of all-cause mortality, with hazard ratios ranging from 1.50 (95% CI 1.14–1.96) to 1.73 (95% CI 1.38–2.16). Dynapenic abdominal obesity was also strongly linked to falls, with pooled estimates ranging from HR = 1.82 (95% CI 1.04–3.17) to RR = 6.91 (95% CI 5.42–8.80). For multimorbidity, older adults with dynapenia had 1.38 times higher odds of having two or more chronic diseases than those without dynapenia (OR = 1.38, 95% CI 1.10–1.72). Based on the GRADE evaluation, the certainty of evidence was moderate for mortality and falls and low for multimorbidity. **Conclusions:** Overall, the findings indicate that dynapenia and its obesity-related forms meaningfully increase the risks of mortality, falls, and multimorbidity among community-dwelling older adults. Importantly, these results position dynapenia not merely as a musculoskeletal condition, but as a clinically relevant marker of aging-related vulnerability. This underscores the need for early screening of muscle strength alongside obesity-related indicators, as well as the development of integrated preventive strategies that combine strength-oriented interventions with obesity management in older populations.

## 1. Introduction

The world’s population is aging at an unprecedented pace, and the number of older adults living with multiple chronic conditions continues to rise. By 2050, the World Health Organization estimates that there will be more than 2 billion adults aged 60 years and older, creating major challenges for health systems and long-term care worldwide [1]. As people age, declines in muscle function become increasingly common. In particular, the progressive loss of muscle strength—known as dynapenia—has gained attention as an important factor linked to disability, dependence, and mortality in later life [2]. Unlike sarcopenia, which emphasizes reductions in muscle mass, dynapenia focuses on the loss of strength even when muscle size is preserved. This shift reflects growing recognition that neuromuscular changes and impaired motor unit activation play critical roles in age-related functional decline [3]. Several studies have also shown that muscle strength predicts mortality more reliably than muscle mass alone, underscoring its clinical importance [4,5]. As a result, dynapenia has emerged as a key concept in geriatric assessment and preventive health care. When dynapenia occurs alongside obesity—referred to as dynapenic obesity—the combined effect of reduced strength and excess adiposity can create an even more harmful health profile. Evidence suggests that individuals with dynapenic obesity face higher risks of metabolic abnormalities, cardiovascular disease, falls, hospital admissions, and premature death [6,7]. The coexistence of muscle weakness and obesity may also intensify low-grade inflammation and insulin resistance, making recovery from illness more difficult [8]. Although several systematic reviews have examined the links between dynapenia, dynapenic obesity, and specific health outcomes, the findings are not always consistent. Differences in study populations, measurement tools, and analytical methods have contributed to mixed conclusions across reviews [9,10], where some report strong associations with mortality and chronic diseases [8,10], while others describe weaker or inconclusive relationships [9,11]. These inconsistencies highlight the need for an umbrella review that brings together evidence from multiple systematic reviews and meta-analyses to evaluate the overall strength and certainty of the existing findings.

To date, no umbrella review has simultaneously examined the relationships among dynapenia, dynapenic obesity, dynapenic abdominal obesity, and major health outcomes such as mortality, falls, and multimorbidity in community-dwelling older adults. Understanding how these conditions interact is essential for improving early screening, guiding health education, and developing interventions that support functional independence. Among older adults living in the community, the combination of muscle weakness and obesity can accelerate physical decline, reduce autonomy, and negatively impact quality of life [11]. By clarifying the evidence, this umbrella review can help inform nursing assessments, resistance training programs, and multidisciplinary strategies aimed at promoting healthier aging.

This umbrella review aimed to comprehensively synthesize and evaluate existing systematic reviews and meta-analyses examining the associations between dynapenia, dynapenic obesity, and adverse health outcomes among community-dwelling older adults. Mortality, falls, and multimorbidity were selected as primary outcomes because they capture complementary and clinically meaningful dimensions of aging-related vulnerability. Specifically, mortality reflects survival, falls represent functional safety and physical stability, and multimorbidity indicates cumulative disease burden. Together, these outcomes provide a life-course- and systems-based perspective on how dynapenia and its obesity-related subtypes influence health trajectories beyond isolated disease-specific endpoints.

## 2. Materials and Methods

### 2.1. Study Design

This umbrella review was conducted following the PRISMA 2020 guidelines and the Joanna Briggs Institute (JBI) recommendations for umbrella reviews [12]. The PRIOR (Preferred Reporting Items for Overviews of Reviews) statement was also consulted to guide reporting and ensure methodological rigor [13]. Because evidence on dynapenia and its obesity-related subtypes is scattered across individual systematic reviews, an umbrella review approach was selected to provide a broad, consolidated understanding of the available findings. The protocol for this review was registered in PROSPERO (ID: CRD 42023415232).

### 2.2. Eligibility Criteria

Studies were eligible if they (1) were systematic reviews or meta-analyses published in peer-reviewed journals; (2) included adults aged 60 years or older or samples with mean age ≥ 60; (3) examined associations between dynapenia, dynapenic obesity, or dynapenic abdominal obesity and adverse health outcomes (e.g., mortality, multimorbidity, and falls); and (4) reported quantitative or pooled estimates (e.g., odds ratio [OR], hazard ratio [HR], relative risk [RR]). Reviews that focused solely on sarcopenia, non-muscle conditions, or narrative summaries without quantitative synthesis were excluded.

### 2.3. Information Sources and Search Strategy

A comprehensive literature search was conducted to capture both English and relevant Chinese-language publications using four bibliographic databases—Embase, Cochrane Library, Scopus, and CINAHL—and two search engines/interfaces, namely PubMed (the search interface for MEDLINE) and Airiti Library. Searches covered all records from database inception to October 2025. Search terms included combinations of “dynapenia,” “muscle strength decline,” “handgrip strength,” “dynapenic obesity,” “systematic review,” and “meta-analysis.” Boolean operators (AND, OR) and relevant MeSH terms were applied. Reference lists of the included reviews were also screened to identify additional eligible studies.

### 2.4. Screening and Selection Process

All retrieved citations were imported into EndNote 21, and duplicates were removed. Two reviewers independently screened the titles and abstracts, followed by full-text assessment based on the eligibility criteria. Any disagreements were resolved through discussion or by consulting a third reviewer to ensure consistency.

**Figure 1:**
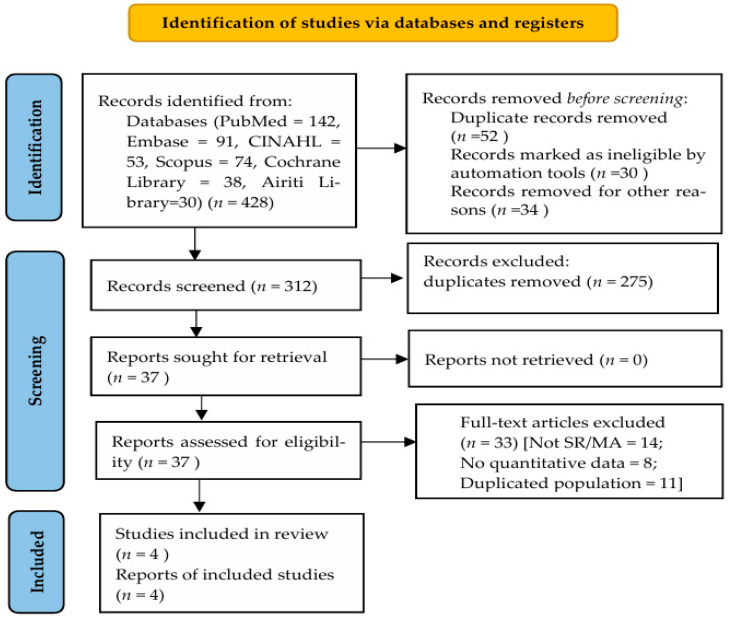
PRISMA 2020 flow diagram of study selection. Records identified in the initial search were excluded due to duplication, non-systematic review design, irrelevant populations or outcomes, lack of quantitative data, or overlapping populations, as detailed at each stage.

Records identified in the initial search were excluded at different stages based on specific exclusion criteria. Duplicate records were removed prior to screening. During title and abstract screening, records were excluded if they were not systematic reviews or meta-analyses, involved populations other than community-dwelling adults aged 60 years and older, or did not examine dynapenia or its obesity-related subtypes. Full-text articles were further excluded if they did not report quantitative or pooled effect estimates, addressed irrelevant outcomes, or represented overlapping populations already included in other reviews. Detailed reasons for exclusion at each stage are presented in the PRISMA 2020 flow diagram (Figure 1).

### 2.5. Data Extraction and Management

A standardized extraction form was developed to collect essential information, including author, publication year, country, sample size, age characteristics, exposure definitions, outcome measures, pooled effect estimates, and statistical models used. Two reviewers independently extracted the data and cross-checked each entry to ensure accuracy. Any discrepancies were resolved through consensus.

### 2.6. Quality Appraisal (AMSTAR 2)

The methodological quality of the included systematic reviews and meta-analyses was assessed using the AMSTAR 2 tool [14]. This instrument evaluates 16 domains such as search adequacy, risk of bias assessment, data synthesis, and reporting clarity. Each domain was rated as “Yes,” “Partial Yes,” or “No,” and overall confidence was categorized as high, moderate, low, or critically low.

### 2.7. GRADE Approach for Assessing Certainty of Evidence

For each key outcome—mortality, falls, and multimorbidity—the certainty of evidence was evaluated using the GRADE framework [15]. Evidence was downgraded or upgraded based on risk of bias, inconsistency, indirectness, imprecision, and publication bias, resulting in classifications of high, moderate, low, or very low certainty.

### 2.8. Data Synthesis

Findings from included reviews were synthesized narratively. Pooled effect sizes, confidence intervals, and the consistency of results across reviews were compared. Overlaps among primary studies were examined to understand redundancy and its potential influence on pooled outcomes. Summary tables were constructed to present the methodological quality, main results, and certainty of evidence for each review.

## 3. Results

### 3.1. Study Selection

The search initially identified 428 records across the six databases. After removing 112 duplicates, 316 titles and abstracts were screened. Among these, 41 full-text articles were reviewed for eligibility. Ultimately, four systematic reviews met the inclusion criteria and were included in this umbrella review. The PRISMA 2020 flow diagram outlines the screening and selection process. Records identified in the initial search were excluded after title and abstract screening if they were duplicates, not systematic reviews or meta-analyses, involved irrelevant populations, or did not report outcomes of interest.

### 3.2. Characteristics of Included Reviews

The four included reviews were published between 2019 and 2023 and together covered more than 73,000 community-dwelling older adults from Asia, Europe, and Latin America. Three were meta-analyses, while one was a systematic review that also incorporated quantitative synthesis. All reviews used established definitions of dynapenia, primarily based on handgrip strength cut-offs recommended by the EWGSOP2 or the Asian Working Group for Sarcopenia (AWGS).

Two reviews focused specifically on dynapenia, whereas the other two examined dynapenic obesity or dynapenic abdominal obesity. Across these reviews, the main outcomes assessed were mortality, falls, and multimorbidity. The follow-up durations varied widely, ranging from short-term assessments to longitudinal cohorts extending up to twelve years. A summary of the review characteristics is presented in Table 1.

### 3.3. Methodological Quality (AMSTAR 2)

Using the AMSTAR 2 checklist, two of the included reviews were rated as high quality, one as moderate quality, and one as low quality. Most reviews had clear inclusion criteria, comprehensive search strategies, and appropriate statistical methods. However, several methodological issues were identified, including the absence of protocol registration in some reviews, limited evaluation of publication bias, and incomplete discussion of potential heterogeneity (Table 2).

### 3.4. Certainty of Evidence (GRADE)

According to the GRADE evaluation, the certainty of evidence was rated as moderate for mortality, and low for falls and multimorbidity. Downgrading was primarily driven by heterogeneity in dynapenia definitions, variability in study designs, and the limited number of available systematic reviews. Despite these limitations, the direction of associations across outcomes was generally consistent (Table 3). In light of potential overlap among reviews and the small number of contributing systematic reviews for certain outcomes, certainty-of-evidence ratings were applied conservatively, and upgrading was avoided where methodological concerns remained. Specifically, the certainty of evidence for mortality was rated as moderate due to consistent effect directions across reviews, relatively precise estimates, and the use of mortality as an objective outcome, whereas no upgrading was applied to falls or multimorbidity because of substantial heterogeneity, limited numbers of contributing reviews, or imprecision.

GRADE definitions:

- High: All domains fulfilled; low risk of bias; consistent findings.
- Moderate: Minor limitations in consistency or precision; overall confidence acceptable.
- Low: Substantial heterogeneity or imprecision; some methodological weaknesses.
- Very Low: Serious limitations in multiple domains; unreliable results.

### 3.5. Mortality in Dynapenic Obesity

Two meta-analyses reported a significant association between dynapenic obesity and all-cause mortality among community-dwelling older adults. The pooled hazard ratios ranged from 1.50 (95% CI: 1.14–1.96) to 1.73 (95% CI: 1.38–2.16), suggesting that older adults with both low muscle strength and obesity had a substantially higher risk of death compared with those without dynapenia or obesity.

### 3.6. Fall Risk in Dynapenic Abdominal Obesity

Two reviews examining dynapenic abdominal obesity found that it was consistently associated with an elevated risk of falls. The pooled effect sizes ranged from HR = 1.82 (95% CI: 1.04–3.17) to RR = 6.91 (95% CI: 5.42–8.80). These findings indicate that the combination of abdominal obesity and reduced muscle strength significantly increases the likelihood of falls among community-dwelling older adults.

### 3.7. Multimorbidity in Dynapenia

One systematic review reported that dynapenia was significantly associated with multimorbidity. Older adults with dynapenia had 1.38 times higher odds of having two or more chronic diseases than those without dynapenia (OR = 1.38, 95% CI: 1.10–1.72). The most common comorbidity clusters involved diabetes, hypertension, and cardiovascular diseases.

## 4. Discussion

The results showed a clear relationship between dynapenic obesity and all-cause mortality in older adults living in the community. Those with both low muscle strength and obesity had a roughly 45–70% higher chance of dying compared with individuals without dynapenia, a pattern that aligns with estimates from earlier meta-analyses. Several biological explanations have been proposed. de Maio Nascimento et al. [16] suggested that muscular weakness combined with excessive fat places a dual metabolic strain on the body. Excess visceral fat promotes persistent low-grade inflammation and insulin resistance through cytokines such as IL-6, TNF-α, and CRP, which contribute to endothelial dysfunction and increased cardiometabolic risk. Muscle weakness associated with dynapenia reflects poorer neuromuscular activation and reduced motor unit recruitment, often occurring alongside myosteatosis, which interferes with glucose handling and further aggravates insulin resistance [17]. Inflammation originating from adipose tissue can also worsen muscle impairment by increasing oxidative stress and mitochondrial injury. Smith et al. [18] pointed out that cross-talk between adipose and muscle cytokines may help explain why dynapenic abdominal obesity is strongly linked to cardiometabolic problems. Taken together, these biological pathways—chronic inflammation, insulin resistance, and muscle deterioration—likely interact to increase the mortality risk observed in individuals with dynapenic obesity.

This umbrella review also identified a clear association between dynapenic abdominal obesity and an increased risk of falls among older adults living in the community. This result is consistent with the findings of de Oliveira Máxim et al. [19] and Liu et al. [10]. In the study by de Oliveira Máxim et al. [19], older adults with dynapenic abdominal obesity were found to have a 60% higher chance of falling compared with those without the condition. The authors explained that this subgroup often exhibits an imbalance between muscle and fat: excess fat raises the mechanical load and energy needed for movement, while insufficient muscle strength weakens postural stability during activities such as standing or walking. This combination restricts mobility and increases vulnerability to falls. Similarly, Liu et al. [10] observed that community-dwelling older adults with dynapenic abdominal obesity were at significantly higher risk of both falls and mortality than those without it. They suggested that reduced muscle strength coupled with abdominal fat accumulation impairs balance and increases physical strain, placing older adults at greater risk for falls and early death. Overall, these observations emphasize that the interaction between muscle weakness and adiposity contributes substantially to fall risk, especially in individuals with compromised postural control and reduced mobility.

This umbrella review also found a clear link between dynapenia and multimorbidity among community-dwelling older adults. Those with dynapenia were more likely to have two or more chronic illnesses compared with individuals without the condition. This pattern was most evident in clusters involving diabetes, hypertension, and cardiovascular disease, suggesting that dynapenia may be an important marker for developing multiple chronic conditions [20]. The relationship likely reflects broader metabolic and inflammatory changes, as declining muscle strength is often a sign of reduced physiological resilience. Persistent low-grade inflammation and metabolic imbalance associated with weak muscle function may encourage the accumulation of chronic diseases and speed up overall health decline, raising multimorbidity risk among older adults with dynapenia. Montes et al. [21] similarly reported that low muscle strength was linked to several chronic illnesses, likely through mechanisms involving inflammation and metabolic dysregulation [18]. Overall, the presence of multimorbidity in individuals with dynapenia appears to stem from interconnected processes involving inflammation, metabolic stress, and neuromuscular deterioration. These mechanisms reinforce each other, making dynapenia a broader indicator of systemic aging rather than a condition limited to muscle strength alone. Therefore, addressing multimorbidity in this population may require a combination of anti-inflammatory dietary strategies, metabolic management, and targeted neuromuscular training to interrupt the cycle of decline.

Accordingly, although the direction of associations across outcomes was largely consistent, the magnitude of pooled estimates and certainty-of-evidence ratings should be interpreted cautiously, particularly in light of the limited number of included reviews and the potential overlap of primary studies across systematic reviews. When overlap was likely, a more conservative interpretation of pooled estimates and certainty ratings was applied, recognizing that such overlap may inflate precision and affect the robustness of the evidence. Furthermore, heterogeneity in handgrip strength cut-offs and measurement protocols across studies—including sex-, ethnicity-, and population-specific thresholds—may have contributed to exposure misclassification and variability in pooled effect estimates, and should be considered when interpreting the magnitude of the observed associations. Although one included review was rated as low quality by AMSTAR 2, the overall conclusions of this umbrella review were primarily driven by findings from moderate- to high-quality systematic reviews. The low-quality review was included for completeness but did not materially influence the direction or interpretation of the main conclusions. Given the limited number of available reviews, a formal sensitivity or credibility-based stratified analysis was not feasible and is therefore acknowledged as a methodological limitation.

### Limitations

This umbrella review has several important strengths. It is the first attempt to bring together evidence on how dynapenia, dynapenic obesity, and dynapenic abdominal obesity are related to major health outcomes in community-dwelling older adults. The review followed strict methodological standards, including PRISMA 2020, PRIOR, and JBI guidelines, and it applied established tools such as AMSTAR 2 and GRADE to evaluate the quality of the included reviews and the certainty of their findings. These approaches help ensure that the conclusions drawn are based on transparent and reliable methods.

There are several limitations that should be acknowledged. First, only four systematic reviews met the inclusion criteria, which may have limited the breadth of the evidence synthesized in this umbrella review. Second, heterogeneity in the definitions and measurement protocols of dynapenia and its related subtypes across studies may have contributed to exposure misclassification. Third, as most included reviews focused on community-dwelling older adults, the generalizability of the findings to institutionalized populations may be limited. In addition, although potential overlap among primary studies was identified across the included systematic reviews, it was not formally quantified using a citation matrix or corrected covered area (CCA). As overlapping evidence may inflate pooled effect estimates and certainty-of-evidence ratings, this methodological limitation should be considered when interpreting the magnitude and certainty of the observed associations. Furthermore, publication bias was not assessed consistently across the included reviews. Despite these limitations, the overall direction of associations across outcomes was generally consistent, lending support to the robustness of the observed findings.

## 5. Conclusions

This umbrella review synthesized current evidence from systematic reviews and meta-analyses examining dynapenia and its obesity-related subtypes among community-dwelling older adults. Overall, the findings suggest that dynapenic obesity and dynapenic abdominal obesity are associated with increased risks of all-cause mortality and falls, while dynapenia alone appears to be linked to multimorbidity. However, the certainty of evidence remains limited by heterogeneity, potential overlap among reviews, and the small number of available high-quality systematic reviews. These findings highlight dynapenia as a potential marker of broader physiological vulnerability in aging, while underscoring the need for future high-quality longitudinal studies to strengthen causal inference.

## Figures and Tables

**Table 1:** Characteristics of included systematic reviews and meta-analyses. DAO, dynapenic abdominal obesity; DO, dynapenic obesity; SR, systematic review; MA, meta-analysis; HR, hazard ratio; RR, relative risk; OR, odds ratio.

Author (Year)	Design	Population	Exposure	Outcomes	Pooled Effect Size (95% CI)	Follow-Up
Liu et al. [10]	MA	13,406 community adults ≥ 60 y	DAO	Fall Mortality	HR = 1.82 (1.04–3.17); HR = 1.50 (1.14–1.96)	2–12 y
Nikkhah et al. [8]	MA	42,000 community older adults	DO	Mortality	HR = 1.73 (1.38–2.16)	2–12 y
Ohinata et al. [5]	SR	6212 community older adults	Dynapenia	Multimorbidity	OR = 1.38 (1.10–1.72)	4–9 y
Kao et al. [9]	MA	11,550 community older adults	DAO	Falls	RR = 6.91 (5.42–8.80)	3–10 y

Definitions of dynapenia, dynapenic obesity, and dynapenic abdominal obesity followed the operational criteria applied in the original systematic reviews and their underlying primary studies. Dynapenia was generally classified based on handgrip strength cut-offs, which varied according to sex-, ethnicity-, and population-specific thresholds. Dynapenic obesity and dynapenic abdominal obesity were defined by the coexistence of low muscle strength and obesity indicators, such as body mass index or waist circumference. Outcome definitions (mortality, falls, and multimorbidity) followed those reported in the included reviews and primary studies.

**Table 2:** Methodological quality of included reviews (AMSTAR 2).

Domain (Critical Domains Noted with **)	Liu et al. [10]	Nikkhah et al. [8]	Ohinata et al. [5]	Kao et al. [9]	No. of Reviews (%) “Yes”
1. Research questions include PICO	Y	Y	Y	Y	4 (100%)
2. Protocol established before review **	Y	PY	PY	Y	2 (50%)
3. Selection criteria explained	Y	Y	PY	Y	3 (75%)
4. Comprehensive literature search **	Y	Y	PY	Y	3 (75%)
5. Study selection in duplicate	Y	Y	N	Y	3 (75%)
6. Data extraction in duplicate	Y	Y	PY	Y	3 (75%)
7. List of excluded studies and justification	Y	Y	N	Y	3 (75%)
8. Description of included studies	Y	Y	PY	Y	3 (75%)
9. Risk of bias (RoB) technique **	Y	Y	N	Y	3 (75%)
10. Sources of funding for included studies **	Y	N	N	Y	2 (50%)
11. Appropriate meta-analysis methods **	Y	Y	N	Y	3 (75%)
12. Impact of RoB assessed	PY	Y	N	PY	1 (25%)
13. RoB considered when interpreting results **	Y	Y	PY	Y	3 (75%)
14. Explanation of heterogeneity	Y	Y	PY	Y	3 (75%)
15. Publication bias investigated **	Y	PY	N	Y	2 (50%)
16. Conflict of interest reported	Y	Y	PY	Y	3 (75%)
Overall confidence	High	Moderate	Low	High	—

Domain (critical domain noted with **); Y (Yes): fully meets the AMSTAR criterion; PY (Partially Yes): partially meets the criterion; N (No): does not meet the criterion; High: no or one non-critical weakness, and the results are highly trustworthy; Moderate: one or more non-critical weaknesses, but no critical flaws, and the results are generally reliable, though some caution is warranted; Low: one critical flaw or multiple non-critical weaknesses, and the results may be affected;

**Table 3:** Certainty of evidence by outcome (GRADE assessment).

Outcome	No. of Reviews	Key Source(s)	Pooled Effect (95% CI)	I^2^	Imprecision	Publication Bias	Certainty of Evidence (Level)
Mortality in Dynapenic Obesity (DO)	2	Liu et al. [10] Nikkhah et al. [8]	HR = 1.50 (1.14–1.96) HR = 1.73 (1.38–2.16)	Moderate (40–55%)	Narrow CIs, large samples	Not detected (funnel plot symmetrical)	Moderate
Falls in Dynapenic Abdominal Obesity (DAO)	2	Liu et al. [10] Kao et al. [9]	HR = 1.82 (1.04–3.17) RR = 6.91 (5.42–8.80)	High (>60%)	Wide CIs in HR study; consistent direction	Possible small-study effect	Low
Multimorbidity in Dynapenia	1	Ohinata et al. [5]	OR = 1.38 (1.10–1.72)	Moderate (45%)	Some imprecision due to sample size	Unclear (no Egger’s test reported)	Low

All included reviews were observational meta-analyses or systematic reviews; therefore, the initial GRADE level was set to Low, with final certainty ratings determined through outcome-specific judgment across relevant GRADE domains.

## Data Availability

No new data were created or analyzed in this study.

## Abbreviations

The following abbreviations are used in this manuscript:

DAO	Dynapenic Abdominal Obesity
DO	Dynapenic Obesity
HR	Hazard Ratio
OR	Odds Ratio
